# The Relationship between Socio-Demographic Factors, Preventive Health Behaviors and Acceptance of COVID-19 Vaccine among Israeli Pregnant Women during the Coronavirus Pandemic

**DOI:** 10.3390/ijerph20156526

**Published:** 2023-08-04

**Authors:** Shir Nahum, Talma Kushnir

**Affiliations:** 1 Department of Psychology, Ariel University, Ariel 40700, Israel; shirnahum7@gmail.com; 2 Adelson School of Medicine, Ariel University, Ariel 40700, Israel

**Keywords:** COVID-19, pregnant women, religious status, socio-demographic factors, preventive health behaviors

## Abstract

Background: The outbreak of the Coronavirus disease led the World Health Organization to publish recommendations regarding preventive health behaviors (PHB). Pregnant women are at a higher risk of severe COVID-19 infection and adherence to these recommendations is critical. There are little data regarding PHB among pregnant women. The current study aims to evaluate the contribution of socio-demographic factors and COVID-19 vaccinations in predicting PHB among pregnant women. Method: 202 pregnant Israeli women (mean age = 30.8 years) participated in an online survey in 2021. Results: 88% of the women were vaccinated and few had been infected. Of the women, 75.2% reported wearing face masks in closed spaces, while 12.4% reported wearing masks outdoors; 63.9% of the women did not travel abroad for fear of infection by the virus and 51% avoided crowded events. A simultaneous regression analysis to predict PHB indicated that pregnancy week and Coronavirus vaccination significantly and positively predicted PHB, but religious status was a negative predictor. Age, number of children, and level of education were not associated with PHB. Conclusions: These findings can be helpful as a preliminary evidence base for policy-making at present and for future epidemics regarding guidelines on PHB adjusted for pregnant women.

## 1. Introduction

Pregnant women are considered at increased risk of adverse outcomes from COVID-19 [1] due to their greater susceptibility to viral infections and altered functioning of the immune system [2]. Adhering to preventive health behaviors (PHB) recommended by global health organizations during pregnancy is therefore important.

Most studies regarding factors that affected adherence to PHB during the COVID-19 pandemic focused on the general population. Only a few focused on pregnant women. Additionally, the existing studies have not examined the effects of the level of religious status and COVID-19 vaccine acceptance on these behaviors. The current study seeks to address these gaps. It examines the contribution of several socio-demographic factors as well as the acceptance of COVID-19 vaccination to adherence to PHB among pregnant women during the pandemic.

### 1.1. Pregnancy during the Coronavirus Pandemic 

Coronavirus disease (COVID-19) is a new respiratory illness caused by the SARS-CoV-2 Coronavirus that was first detected in 2019 in Wuhan, China and spread rapidly worldwide. The virus spreads by being in contact with infected droplets and surfaces, causing a disease that affects the respiratory tract [3,4,5]. To date, over 680 million people have been infected with Coronavirus and nearly 7 million have died [6].

Medically, pregnant women may be more vulnerable to respiratory infections, including those caused by the Coronavirus, compared to the general population. This is due to immunological and physiological changes that occur in their bodies during pregnancy [7]. Indeed, pregnancy has been considered a predisposing factor to severe COVID-19 [8,9,10,11]. Pregnant women with COVID-19 have higher chances of being admitted to the intensive care unit and of maternal death. Furthermore, they have a higher risk of preterm delivery and stillbirth when compared to pregnant women without COVID-19 [8,12]. 

The Coronavirus pandemic can affect the mental well-being and quality of life of pregnant women. Pregnant women during the Coronavirus pandemic showed more pronounced increases in depression and anxiety than non-pregnant women [13] and as compared to similar pre-pandemic pregnancy groups [14]. In addition, they reported high levels of pregnancy-related worry during this period [11,15]. High levels of worry and anxiety during the Coronavirus pandemic were found to be related to several factors, such as fear of infection, fear of changing birth plans, lack of face-to-face social interaction, the perception of possible future negative consequences [11], and concern for the well-being and health of the fetus [15,16]. The exact effects of the new virus on the health of the fetus are still unknown, which can create great uncertainty among pregnant women [10,17]. 

### 1.2. Health Behaviors among Pregnant Women

Health behaviors are a number of behavior patterns that people perform to maintain and promote their physical and mental health [18]. The World Health Organization recommend that pregnant women perform a variety of health behaviors in order to maintain their health and the normal development and growth of the fetus, such as by using nutritional supplements, maintaining a proper diet, avoiding smoking and drinking alcohol, and reducing caffeinated beverages [19,20]. Pregnant women reported that they performed more health behaviors during pregnancy than the period before it, including a decrease in the level of smoking and a reduction or cessation of the consumption of alcoholic beverages and beverages containing caffeine. However, there seems to have been no real change in women’s consumption of vegetables and fruits compared to the period before pregnancy [21].

Due to the spread of the COVID-19 virus, world health organizations formulated various recommendations for the prevention of the viral infection and the treatment of the disease [22,23,24]. The recommendations vary according to the area and the outbreak of the virus. They include the wearing of masks, the use of disinfectants, testing and isolation for people who have been infected, restrictions on flights to destinations abroad, and the prevention of social gatherings [25,26]. These guidelines included the general population and pregnant women [25].

Studies conducted during the Coronavirus pandemic found that pregnant women often perform PHB due to COVID-19 [27,28]. For example, in a study conducted in Colorado among pregnant women, it was found that all of the women reported changes in their health behaviors during the Coronavirus pandemic, which included increasing the frequency of hand washing, frequent use of antiseptic gel, reducing meetings with friends (social distancing), avoiding people who had symptoms of Coronavirus, using masks, and avoiding touching the face, nose, and mouth [29].

In addition, as part of promoting the health of pregnant women, health organizations currently recommend that pregnant women get vaccinated against the Coronavirus due to encouraging findings on the success of vaccinations [30,31,32,33]. However, there is limited responsiveness among pregnant women [34,35], perhaps because of concerns about the effect of vaccination on the health of the fetus and the woman [36], as well as the numerous side effects that occur after receiving the vaccine [37]. It should be noted that pregnant and lactating women were often not included in the clinical trials conducted to develop vaccines during the epidemic, which can make it difficult to draw conclusions for this population [38,39].

It has long been recognized that there are variations in pregnant women’s attitudes toward health care provider recommendations. There are women who carefully follow treatment plans and receive the required medical treatment as is; on the other hand, there are women who choose not to accept the recommendations accurately and change them accordingly when they feel they are not beneficial for them [40,41,42]. Specifically, it is known that socio-demographic factors and socio-cultural contexts may influence decision-making regarding the PHBs of pregnant women against the Coronavirus [27,43]. Therefore, it is important to examine the contribution of such background factors in predicting PHB against the Coronavirus among pregnant women.

### 1.3. Socio-Demographic Factors and Health Behaviors against the Coronavirus

Previously, many studies have focused on socio-demographic factors that influence PHB in the general population. New studies since the outbreak of the Coronavirus pandemic have focused on factors related to health behaviors against the Coronavirus. For example, it appears that older adults engage in PHBs against the Coronavirus more than younger adults [44]. Gender and income level also influence health behaviors so that women tend to perform more PHBs than men, and people with a higher income level perform more PHB than people with a lower income level [45]. Another factor that can influence health behavior is marital status, as married people have been found to perform more PHBs than single people [46].

The role of socio-demographic variables in negative health behaviors has also been studied. For example, in a study conducted in Canada at the beginning of the Coronavirus outbreak, it was found that young adults are more likely to engage in negative health behaviors such as alcohol consumption, junk food consumption, and increased screen time, than older adults [47]. 

Only few studies have focused on the population of pregnant women [48,49,50]. In a study that examined PHB against the Coronavirus, psychological health, and socio-demographic variables among pregnant women, it was found that among the socio-demographic variables studied (age, education, week of pregnancy, marital status, employment, and the number of children at home), only educational level was found to be related to PHB. Thus, women with a higher level of education performed more PHBs relative to women with a lower level of education [51]. However, the study sample included only 35 pregnant women. The current study included over 200 women and therefore may contribute additional information. Another study examined the relationship between age, educational level, occupation, socio-economic status, and marital status in predicting PHB among pregnant women visiting prenatal clinics in the Obio/Akpor Local Government Area of Rivers State. The findings indicate that, among these variables, only the women’s ages significantly predicted PHB [50].

Another important socio-demographic factor is religiosity. Religious status is a behavioral aspect that gains increased importance in times of distress. For example, research during the Coronavirus pandemic revealed a significant increase in searches for prayers in about a hundred countries, even in countries with a very secular character [52]. However, only few studies have examined the relationship between religious status and PHB among pregnant women. Recently, Abd Elaziz [53] compared the differences in performing PHB among Muslim and Christian pregnant women and found no differences in their behaviors. However, the study sample did not include secular women. The current study that included secular participants may add important information about the role of religiosity level in predicting PHB. 

Maintaining social distancing and avoiding gatherings were among the preventive behaviors against COVID-19 that were recommended by health organizations [4]. However, social distancing is contrary to a religious lifestyle that is usually characterized by joint gatherings and close human contact, especially during prayers and worship. It is therefore important to compare secular and non-religious women and religious women in studies of socio-demographic details that predict engagement in recommended PHB during COVID-19. The current study that includes both secular and religious women may add important information about the role of level of religiosity in predicting PHB. 

Another gap in studies conducted during the Coronavirus pandemic is the lack of attention given to the possible association between vaccination against Coronavirus and other PHBs among pregnant women. Due to encouraging findings about the successes of vaccines and the feeling that they may provide protection against the infection [30], it can be assumed that women who had been vaccinated against the Coronavirus at least once performed fewer PHBs than women who had not been vaccinated at all. 

Geographical location may also be an important related factor that may affect PHB because the severity and spread of diseases may differ among countries. Support for this possibility can be found in the study by Pope [27], who found that the country and place of residence affect health behaviors of pregnant women. American women reported a higher frequency of mask-wearing than women from the United Kingdom and Ireland. In addition, residents who live in rural areas are less likely to wear a mask than urban residents [54]. There has not yet been a study of the contribution of socio-demographic factors among Israeli pregnant women.

In sum, there is a paucity of studies regarding the effects of socio-demographic factors and COVID-19 vaccines on engagement in PHB among pregnant women during the Coronavirus pandemic and none have been conducted in Israel. The current study seeks to examine the association between several socio-demographic factors (age, educational level, week of pregnancy, and level of religiosity) and acceptance of COVID-19 vaccines, and PHB among pregnant Israeli women.

The results may help health policymakers understand the factors influencing PHB during the COVID-19 pandemic in the population of pregnant women who are at risk of infection and draw conclusions in preparation for future epidemics. Therefore, the hypotheses in the present study are:

- Performance of preventive health behaviors will be positively associated with the woman’s age, week of pregnancy, and educational level.
- Religious women will perform fewer PHBs than secular women.
- Women who have not been vaccinated against the Coronavirus will perform more PHBs than women who were vaccinated once or twice.

## 2. Materials and Methods

### 2.1. Sample and Sample Size Determination

The sample included 202 Israeli women who were pregnant between September and November 2021 (during the Delta period, June–December, 2021), between two significant outbreaks of the Coronavirus in Israel. All of the women were over the age of 18. The sample size was determined based on statistical considerations and research objectives. In this study, we referred to a population of 182,016 pregnant women, according to the Central Bureau of Statistics in Israel [55]. To achieve a 7% margin of error (MOE = 0.07), 95% confidence level, and response distribution of 50%, we surveyed 200 participants using random sampling. This sample size allows us to draw meaningful conclusions about the characteristic of interest within the population.

### 2.2. Research Tools

- Demographic data included age, four levels of education (secondary–PhD), extent of employment, number of children at home, and level of religiosity (four levels: secular, traditional, Orthodox, ultra-Orthodox). Two items were pertaining to pregnancy: week of pregnancy and whether the pregnancy was planned or not.
- Four questions about Coronavirus: infected with the Coronavirus, vaccinated against COVID-19, time of vaccination (before or during pregnancy), and the number of vaccinations received.
- Nine questions about preventive behaviors from COVID-19. The items were taken from the studies of Shahnazi and Aschwanden [56,57] and adapted to the current study according to the latest recommendations for the months between August and November, 2021 issued by the Israeli Ministry of Health [25]. Responses to the questions about PHB among pregnant women during the Coronavirus disease included 5-point Likert scales, where 1 represented “Never”; 2 denoted “Rarely”; 3 indicated “Sometimes”; 4 represented “Very often”, and five “Always”. The higher the total score, the higher the PHB performed during the COVID-19 disease. Sample questions: “I wear a face mask in closed places\outdoors”; “I use hand sanitizer often”; “I keep a proper distance from others”. Cronbach’s alpha reliability of the questionnaire in the current study is α = 0.862.

### 2.3. Procedures

The study was approved by the Institutional Ethics Committee of Ariel University. The study design included a convenience sampling method and an online survey with a structured questionnaire utilizing social media platforms, such as Facebook and WhatsApp social network groups and channels. Convenience sampling was chosen due to its practicality and ease of access to potential participants, especially during social distancing. In order to reach a diverse group of pregnant women, the researchers strategically reached out to various online communities and forums relevant to Israeli expectant mothers. Also, posts were published targeting pregnant women living in urban and rural areas and holding different levels of religiosity. These outreach efforts ensured that the recruitment notice reached a diverse audience of pregnant women from different cultural and religious backgrounds and geographical locations. The participants responded to the questionnaires using Qualtrics software. All participants in the study provided informed consent by digitally signing an online consent form.

In this study we did not conduct a pilot test. However, in the process of compiling the questionnaire, we presented the items to several colleagues as well as pregnant acquaintances and then amended the items according to the opinions they expressed regarding the clarity, comprehensibility, and length of the questionnaire. In addition, during data collection, the leading researcher’s email was available to the participants for queries and comments regarding the study and the questionnaire.

### 2.4. Statistical Analyses

Several preliminary analyses were performed before examining the research hypotheses. First, a reliability analysis was performed for the research questionnaires. After that, descriptive statistics were used to summarize information for all of the variables in the study. Subsequently, Pearson correlations were calculated between all of the quantitative variables and PHBs. All analyses were conducted using SPSS version 25. A simultaneous multiple regression analysis was conducted to examine the relationship between PHB and age, the week of pregnancy, the number of vaccinations against the virus, the level of religiosity, and years of education. The analysis included two dummy variables that were subjected to RECODE statistical manipulation. The first was the religious status and was coded according to two groups: 1: secular women/non-religious *n* = 83) and 2: religiosity level (traditional, Orthodox, and ultra-Orthodox) (*n* = 119). The second variable is COVID-19 vaccination history, which was coded according to two groups: 1—women without vaccination (*n* = 24) and 2—women who were vaccinated at least once (*n* = 178).

In this study, the assumption of normality was assumed due to the substantial sample size of *n* = 202. This decision was based on the Central Limit Theorem, which posits that, with a sufficiently large sample size, the distribution of sample means tends to approximate a normal distribution, regardless of the underlying population distribution.

## 3. Results

### 3.1. Descriptive Statistics of Socio-Demographic and Coronavirus Characteristics among Pregnant Women

Table 1 showed that the average age of the women was 30.8. Most of the women in the sample (58.9%) were religious to some extent. Slightly more than half of the women in the sample (53.5%) were pregnant for at least the second time. Most of the women had an academic education (85.1%), and most of them worked full-time while being pregnant (78.2). Most of the women were not infected with the Coronavirus (90%). Of the women who were infected, half became sick before pregnancy (5%) and half got sick during pregnancy (5%). In addition, most women had been vaccinated against the Coronavirus: 3.5% once, 30.7% twice, and 54% 3 times. Only a few were not vaccinated at all (11.9%). Vaccination occurred more often during pregnancy (54.5%) than before pregnancy (33.7%).

### 3.2. Preventive Health Behaviors

Table 2 shows that the most common behavior among pregnant women was wearing a face mask in closed places, with 75.2% of the women reporting that they wore a face mask “always” or “very often”. The least common behavior was wearing a face mask outdoors (12.4% of women). Furthermore, most women (62.8%) “rarely” or “never” used hand sanitizer but about half of them (51%) reported trying to avoid going to parties and crowded events and avoiding travel abroad for fear of getting infected (63.9%). 

### 3.3. Hypotheses Testing

To investigate which predictors uniquely explained the variation in PHB among Israeli pregnant women, the continuous predictors and dummy predictors were entered into a simultaneous regression. In conducting the linear regression analysis, we assessed the assumption of linearity between the independent variable and the dependent variables. Visual examination of scatterplots and statistical tests did not reveal any deviations from linearity. This model explained 12.1% of the variance in PHB, F(5, 196) = 5.39, *p* < 0.001. The age and level of education did not significantly predict PHB, whereas week of pregnancy, number of COVID-19 vaccinations, and level of religiosity were significant predictors; thus, the results indicate that an advanced pregnancy week predicts more preventive behaviors. Also, women who had received one or more COVID-19 vaccines performed more PHBs than women who had not been vaccinated at all. Finally, secular women performed more PHBs than religious women. Table 3 indicates the standardized regression coefficients of the predictors in the simultaneous regression model.

## 4. Discussion

The current study investigated the effects of socio-demographic characteristics and COVID-19 vaccine acceptance on preventive health behaviors among pregnant Israeli women during the COVID-19 pandemic. The study was conducted between two major outbreaks of the Coronavirus in Israel. In the current sample, among all the preventive health behaviors reviewed, wearing a face mask in closed places was the most common behavior, in support of Moradi et al.’s research [48]. Wearing a face mask outdoors was the health behavior that the pregnant women observed to the least extent relative to the other behaviors.

In addition, the results indicate that women who had not received COVID-19 vaccinations at all performed fewer PHBs compared to women who had received a vaccination at least once. This finding is contrary to the research hypothesis. It is possible that the finding reflects the phenomenon of COVID-19 denial, when people do not believe that the Coronavirus really exists or who support conspiracy theories and therefore oppose receiving vaccines and avoid following the recommendations of health organizations [58]. Thus, it is possible that some of the women in the present study sample were amongst COVID-19 deniers and felt less need to adhere to the guidelines recommended by health organizations. Another possible denial explanation is related not to the existence of the disease but rather to the denial of its personal relevance, i.e., a vulnerability denial [59] or underestimation of the consequences of infection. Future studies should examine the scope of the denial phenomenon among pregnant women. In addition, it is important that future studies examine the timing of infection with the Coronavirus among large samples. In the current study, only 20 women were infected with the Coronavirus before or during pregnancy, which made it difficult to draw meaningful statistical conclusions. 

The present findings indicate that, as expected, the level of religiosity negatively predicted PHB, so that religious women (traditional, Orthodox, or ultra-Orthodox) reported that they performed fewer preventive behaviors than non-believing/secular women. This finding may reflect cognitive as well as cultural differences that underlie the few existing studies. Firstly, it may reflect the fact that religious women perceived God as all-powerful and that “He” is the determiner of one’s fate and/or the belief that “God is the shield” and therefore they might have felt more protected from the consequences of the Coronavirus than non-believers [60,61]. An alternative explanation reflects differences in cultural practices related to the level of religiosity. It could be that the results reflect the way information is disseminated and messages are conveyed among ultra-Orthodox populations who discourage the use of social networks and TV-watching [62], which were central tools for the dissemination of messages regarding health behaviors during the Coronavirus period by health organizations. It was recently found that users of social media were about three times more likely to follow PHBs than those who avoid social media [63,64]. However, there are few existing studies to substantiate this possibility and further research may shed light on this issue. Another explanation that warrants further investigation is that religious women may tend to follow and trust recommendations issued by religious leaders such as priests and rabbis who influence the women more strongly than health authorities, yet religious authorities may not be scientifically updated.

In an attempt to reach out to religious communities so as to increase compliance with official recommendations on preventive measures, the President of Israel, Isaac Herzog, convened the heads of Christianity, Islam, Baha’is, Druze, and the two chief rabbis of Israel in a first-of-its-kind meeting at his residence in 2020. In this major attempt at enlisting religious leaders to form a joint action in the fight against COVID-19, he called for interfaith cooperation aiming to increase compliance with vaccination policies. During this professional conference with the participation of doctors and religious leaders of all denominations, ways of persuading COVID-19 anti-vaxxers were also presented. It is important to make additional efforts to increase responsiveness among religious pregnant women [65] and this example of enlisting religious leaders in health communications may signal a venue for making such efforts. 

Another interesting finding of this study is that the week of pregnancy significantly affected preventive health behaviors, so that women who were in a more advanced week of pregnancy performed more PHBs. It is possible that the more advanced the women are in their pregnancy, the more they are concerned about their health and the fetus’s health and therefore perform more health behaviors. This hypothesis should be tested in future studies. It is important to emphasize that the effects of the infection of a pregnant woman by the Coronavirus on the health of the fetus are still not completely known, which can create uncertainty among pregnant women [10,17]. The uncertainty and fear can motivate pregnant women to perform preventive behaviors, which is in accordance with studies that show the connection between negative emotional reactions such as fear, worry, and anxiety and PHB against the Coronavirus [66,67,68]. Further studies should examine the level of concerns regarding the fetus’s health as a mediating variable between the week of pregnancy and the performance of PHBs. Another explanation is that women with advanced pregnancy performed more PHBs as they feared getting infected by Coronavirus and the possibility of delivering alone in COVID departments without the presence of family members and the partner, due to the restrictions imposed by social distancing.

Contrary to the research hypothesis, the age of the pregnant women in this sample was not found to have a significant contribution to the prediction of PHB, as opposed to findings in the general population [44]. However, this finding is consistent with the reports by Abd Elaziz [53] that age and PHB were not associated among pregnant women. Nevertheless, the study by Besho [49] did find a significant relationship between the age of the pregnant woman and PHB, with women who were 25 years of age or younger performing more health behaviors. Additional studies on the subject of age in a representative population are warranted. 

In summary, the present study found that the week of pregnancy and the number of COVID-19 vaccinations were positively related to PHB, while the level of religiosity was a negative predictor. Age, extent of employment, and the level of education were not associated with PHB.

### Study Limitations

This study has several limitations. First, it did not include the residential area of the pregnant women. In other studies, a significant relationship was found between the place of residence (rural/urban) and PHB [49], a factor that can help understand the contribution of socio-demographic variables to health behavior during pregnancy. Second, since the study only included Jewish participants, it is recommended that studies on PHB include participants from other faiths/religions so as to increase the variance explained in the research model. Additionally, the present study employed online recruitment as the primary method for participant recruitment. However, it is crucial to acknowledge that the use of convenience sampling introduces a potential of research bias. The results obtained from convenience samples may not accurately represent the broader population, raising concerns about external validity. This can be seen in the high level of education reported by the women included in the study; therefore, it is not possible to determine if the level of education was not related to the use of PHB. In addition, it is important to note that the variance explained in the model was found to be rather low, with the week of pregnancy indicating the highest predictability in the model. Therefore, apart from socio-demographic variables, there may be additional factors that influence the performance of PHB among pregnant women in this sample. Such factors may include personality variables such as optimism and conscientiousness, knowledge and awareness of protective guidelines against the virus, health status, and access to health services.

Despite these limitations, the findings may help healthcare policymakers focus on guidelines for preventing COVID-19 infections among pregnant women and for preparing for future epidemics. The population of pregnant women is not usually included in clinical trials conducted to develop vaccines, and information about them is limited [38,39]. Therefore, it is of particular importance to draw empirical conclusions regarding this population in preparation for possible future epidemics in order to try to increase women’s responsiveness in performing PHBs. Accessing knowledge and tailored education for this population may help them maintain and promote their health and the health of their fetuses.

## 5. Conclusions

Preventive health behaviors were explained by pregnancy week, religious status, and COVID-19 vaccine acceptance. The findings reveal new knowledge regarding the importance of the level of religious status and vaccine acceptance in predicting preventive health behaviors among pregnant women. These findings can be helpful in serving as preliminary data for the specific targeting of interventions as well as policy-making and guidelines on recommended health behaviors during epidemics, adjusted for pregnant women.

The most meaningful finding with practical implications is the positive association between the week of pregnancy and PHB. It suggests that recommendations for health promotion and preventive behaviors should be presented to pregnant women by relevant healthcare authorities as early as possible. The finding that religious women performed less PHBs than secular women underscores the importance of enlisting cooperation between religious leaders and public health authorities, especially during a pandemic. 

## Figures and Tables

**Table 1:** Descriptive statistics of socio-demographic and Coronavirus characteristics (*n* = 202).

Socio-Demographic Characteristics	*n*	*%*	*M*	*SD*
Age			30.76	5.06
Week of pregnancy			25.03	9.32
Educational level				
High school education	30	14.9		
BA	105	52		
MA	60	29.7		
PhD	7	3.5		
Religiosity level				
Secular	83	41.1		
Traditional	30	14.9		
Orthodox	80	39.6		
Ultra-Orthodox	9	4.5		
Number of children at home				
0	94	46.5		
1	49	24.3		
2	37	18.3		
3	15	7.4		
4+	7	3.5		
Work during pregnancy				
Full-time	158	78.2		
Unemployed	23	11.4		
Part-time	21	10.4		
Planned pregnancy				
Yes	177	87.6		
No	21	10.4		
Irrelevant	4	2		
Infection with the Coronavirus				
No	182	90		
Before pregnancy	10	5		
During pregnancy	10	5		
COVID-19 Vaccine history				
Not vaccinated	24	11.8		
One vaccine	7	3.5		
Two vaccines	62	30.7		
Three vaccines	109	54		
Time of vaccination				
Not vaccinated	24	11.8		
Before pregnancy	68	33.7		
During pregnancy	110	54.5		

**Table 2:** Frequency and percentage of COVID-19 preventive health behaviors (PHBs) by pregnant women (*n* = 202).

Preventive Behaviors against COVID-19	Never	Rarely	Sometimes	Very Often	Always
1. I keep a proper distance from others	9 (4.5)	44 (21.8)	90 (44.6)	49 (24.3)	10 (5)
2. I use hand sanitizer often	72 (35.6)	55 (27.2)	27 (13.4)	32 (15.8)	16 (7.9)
3. I avoid entering the elevator when other people enter	42 (20.8)	37 (18.3)	69 (34.2)	40 (19.8)	14 (6.9)
4. I wear a face mask in closed places	2 (1)	16 (7.9)	32 (15.8)	52 (25.7)	100 (49.5)
5. I wear a face mask outdoors	58 (28.7)	65 (32.2)	54 (26.7)	18 (8.9)	7 (3.5)
6. I follow the instructions from the health organizations and act according to them	18 (8.9)	28 (13.9)	54 (26.7)	71 (35.1)	31 (15.3)
7. I avoid going to parties and crowded events for fear of getting infected	23 (11.4)	29 (14.4)	47 (23.3)	54 (26.7)	49 (24.3)
8. I avoid going to events with many participants for fear of getting infected	23 (11.4)	30 (14.9)	55 (27.2)	55 (27.2)	39 (19.3)
9. I avoid traveling abroad for fear of getting infected	28 (13.9)	21 (10.4)	24 (11.9)	40 (19.8)	89 (44.1)

**Table 3:** Simultaneous regression to predict PHB by age, week of pregnancy, number of COVID-19 vaccinations, level of religiosity, and level of education among pregnant women (*n* = 202).

	B	Standardized β	Std. Error	t	*p*
Age	0.00	0.01	0.01	0.07	0.944
Week of pregnancy	0.02	0.24	0.01	3.46	0.001
Level of education	0.13	0.12	0.08	1.72	0.086
Number of COVID-19 vaccinations	0.36	0.14	0.17	2.06	0.041
Level of religiosity	−0.26	−0.16	0.13	−2.05	0.041

Note: R^2^ = 0.12, F (5, 196) = 5.39, *p* < 0.001.

## Data Availability

Data are available on request from Shir Nahum.
